# Prognostic and clinical significance of tumor-associated macrophages in esophageal squamous cell carcinoma after surgery: do biomarkers and distributions matter?

**DOI:** 10.1042/BSR20231194

**Published:** 2024-04-04

**Authors:** Bin Yi, Jun Zeng, Linfeng Li, Junjie Zhang, Yufan Chen, Yang Gao

**Affiliations:** 1 Department of Thoracic Surgery, Xiangya Hospital, Central South University, Xiangya Road 87th, Changsha, 410008 Hunan, P.R. China; 2 Xiangya Lung Cancer Center, Xiangya Hospital, Central South University, Changsha,410008 Hunan, P. R. China; 3 National Clinical Research Center for Geriatric Disorders, Changsha, 410008, Hunan, P.R. China

**Keywords:** Clinical significance, Esophageal squamous cell carcinoma, Pooled analysis, Prognostic significance, Tumor-associated macrophages

## Abstract

Background: The role of tumor-associated macrophages (TAMs) in patients with esophageal squamous cell carcinoma (ESCC) following surgery remains controversial. Hence, we performed the present study to systematically analyze the prognostic and clinical significance of distinct TAMs biomarkers and distributions in ESCC patients underwent surgery.

Methods: PubMed, Web of Science, and EMBASE databases were searched up to March 31, 2023. The pooled analysis was conducted to evaluate the effects of TAMs on overall survival (OS), disease-free survival (DFS), and clinicopathological characteristics using fixed-effects or random-effect model.

Results: Involving a total of 2,502 ESCC patients underwent surgery from 15 studies, the results suggested that the total count of CD68+ TAMs was inversely associated with OS and DFS in ESCC patients, which was also noticed in the relationship of CD68+ TAMs in tumor islet (TI) with OS (all *P*<0.05), although no association between CD68+ TAMs in tumor stroma (TS) and OS (*P*>0.05). Moreover, either islet or stromal CD163+ TAMs density was a prognostic factor ESCC (all *P*<0.05). Similarly, an elevated CD204+ TAMs density in TI predicted a poor DFS (*P*<0.05), although CD204+ TAMs in TI had no relationship with OS (*P*>0.05). Besides, a high CD68+ TAMs density was significantly associated with lymphatic vessel invasion, vascular invasion, and lymph node metastasis (all *P*<0.05).

Conclusion: Our results demonstrated the prognostic and clinical significance of TAMs in ESCC patients underwent surgery. TAMs should be considered a target that could improve prognostic stratification and clinical outcomes in ESCC after surgery.

## Introduction

Esophageal carcinoma, one of the most common malignant gastrointestinal carcinomas, is ranked seventh globally in terms of incidence, with a staggering 604,000 new cases [1]. Despite the introduction of novel therapeutic strategies such as video-assisted thoracoscopic surgery and molecularly targeted therapy, the 5-year survival rate for esophageal carcinoma remains disappointingly unchanged, particularly for individuals with advanced-stage diseases [2]. In 2020, esophageal carcinoma has the sixth-highest mortality among malignancies, which is responsible for 544,000 deaths in the world [1]. Consequently, it becomes imperative to explore biomarkers that can effectively reflect the tumor's biological behavior and prognosis in esophageal carcinoma.

The tumor microenvironment, consisting of various immune cells, plays an important role in tumor progression, invasion and metastasis of tumors [3,4]. Tumor-associated macrophages (TAMs), a class of immune cells, constitute approximately 50% of all cells and are a major component in the tumor microenvironment of solid tumors [5]. TAMs exert significant influence on numerous aspects of tumor cell biology, encompassing antigen presentation, angiogenesis, tissue repair, and tumor cell destruction [6]. Prior research suggests that TAMs drive pathological processes and possess prognostic significance in diverse cancers, including lung cancers [7], liver cancers [8], and breast cancers [9]. In general, a high infiltration of TAMs in tumor microenvironment indicates a poor prognosis, however, outcomes vary depending on macrophages biomarkers and histologic locations in patients with esophageal carcinoma [10–12]. Esophageal adenocarcinoma is the predominant subtype of esophageal carcinoma in North America and Europe, whereas esophageal squamous cell carcinoma (ESCC) is the most common subtype in China, accounting for over 90% among esophageal carcinoma patients [13]. Up to now, ESCC remains the primary subtype among esophageal carcinoma cases. Consequently, we present the first comprehensive study to assess the prognostic and clinical significance of distinct macrophage biomarkers and tissue distributions of TAMs in the tumor microenvironment of ESCC.

## Methods

### Literature search‘

This study was registered with PROSPERO (CRD42022324113). In line with the Preferred Reporting Items for Systematic Reviews and Meta‐Analyses (PRISMA) guidelines [14], we conducted a comprehensive search of PubMed, Web of Science, and EMBASE databases to identify potential studies published in scholarly journals up until March 31, 2023. The Medical Subject Heading terms and/or text words entered were ‘macrophage’, ‘tumor-associated macrophage’, ‘esophageal’, ‘esophagus’, and ‘oesophagus’. We also undertaken forward and backward citation tracking to identify additional non-indexed literature. No language or country restrictions were applied to the present pooled analysis. Titles and abstracts of the articles obtained from these searches were independently screened by two reviewers to ascertain if they met the inclusion criteria.

### Inclusion and exclusion criteria

We included studies reporting TAMs associated with ESCC. To be considered for inclusion, studies had to meet the following criteria: (1) they were cohort studies, such as prospective, retrospective, or case control studies. (2) TAMs must be measured at the primary tumor site using immunohistochemistry with the markers including CD68, CD163, or CD204. (3) patients were diagnosed as ESCC through pathology; and (4) the studies reported the association of TAMs with overall survival (OS), disease-free survival (DFS), and clinicopathological characteristics.

The following studies were excluded: (1) those that fell into specific types of literature, including reviews, comments, and conference abstracts; (2) TAMs were measured at metastatic or local relapse sites; (3) patients with esophageal carcinoma were diagnosed with non-ESCC, such as esophageal adenocarcinoma; and (4) studies that did not provide relevant results or had repetitive raw data.

### Data extraction and quality assessment

Referring to the inclusion and exclusion criteria, relevant data were extracted independently from the original studies by two reviewers. Discrepancies would be resolved by re-evaluation and discussion with the other reviewer. The following indices were collected from each study: the last name of the first author, year of publication, demographic characteristics of participants, macrophage markers, macrophage distribution (tumor islet [TI] or tumor stroma [TS]), tumor stage, OS and DFS with hazard ratios (HRs) and 95% confidence interval (CI). In addition, we collected the prognostic information from study only reported with a Kaplan–Meier (KM) plot and a *P-*value derived from log-rank analysis. HRs and 95%CI were extracted from KM plot using Engauge Digitizer version 4.1 (a freely available software downloaded from http://sourceforge.net) and calculated as previously described [15]. When necessary, authors were contacted for additional unpublished data.

Two experienced reviewers independently assessed the quality of each included study using the modified Newcastle–Ottawa Scale (NOS) [16]. Studies were scored in accordance to three evaluation indexes, including patient selection, study comparability and outcome assessment. The included study was graded as high quality with an NOS score ≥ 6. Disagreements were resolved by a third reviewer.

### Statistical analysis

The statistical analysis was conducted following the guidelines set forth by The Cochrane Collaboration. We employed hazard ratios (HRs) with a 95% confidence interval (CI) to assess the relationship between TAMs (tumor-associated macrophages) density and survival outcomes. To examine dichotomous data related to clinicopathological features, we utilized odds ratios (OR) along with their corresponding 95% CI. Our findings were depicted graphically using either a forest plot or a table. To evaluate variations within and between studies, we employed Cochran’s Q-statistics. Additionally, we assessed heterogeneity across studies using the *I*^2^ statistics, which range from 0 to 100%. For *I*^2^ values below 50%, indicating low heterogeneity, we conducted data analysis using a fixed-effect model. Conversely, for high heterogeneity with *I*^2^ values greater than or equal to 50%, we employed a random-effect model. Sensitivity analysis was performed by altering the statistical method and analysis model.

The statistical analysis was performed according to the recommendations from Cochrane Collaboration. The HRs with 95% CI was used to evaluate the correlation between the TAMs density and survival outcomes. The odds risk (OR) and corresponding 95% CI for the difference in clinicopathological features were used to measure the dichotomous data. Results are presented graphically using a forest plot graph or table. Cochran’s Q-statistics was used to evaluate within- and between-study variations. Heterogeneity across studies were assessed by the *I*^2^ statistics, which ranged from 0 to 100%. Data were analyzed with a fixed-effect model for *I*^2^ < 50%, which was considered as low heterogeneity. Otherwise, the random-effect model was applied for high heterogeneity with *I^2^* ≥ 50%. Sensitivity analysis was conducted by shifting the statistical method and analysis model. Potential publication bias was assessed by funnel plots, when the number of included studies reached five or more. The pooled data were analyzed using Review Manager Version 5.3 software (The Nordic Cochrane Center, The Cochrane Collaboration, 2014, Copenhagen). *P*<0.05 was considered statistically significant.

## Results

### Search results and study characteristics

**Figure 1:**
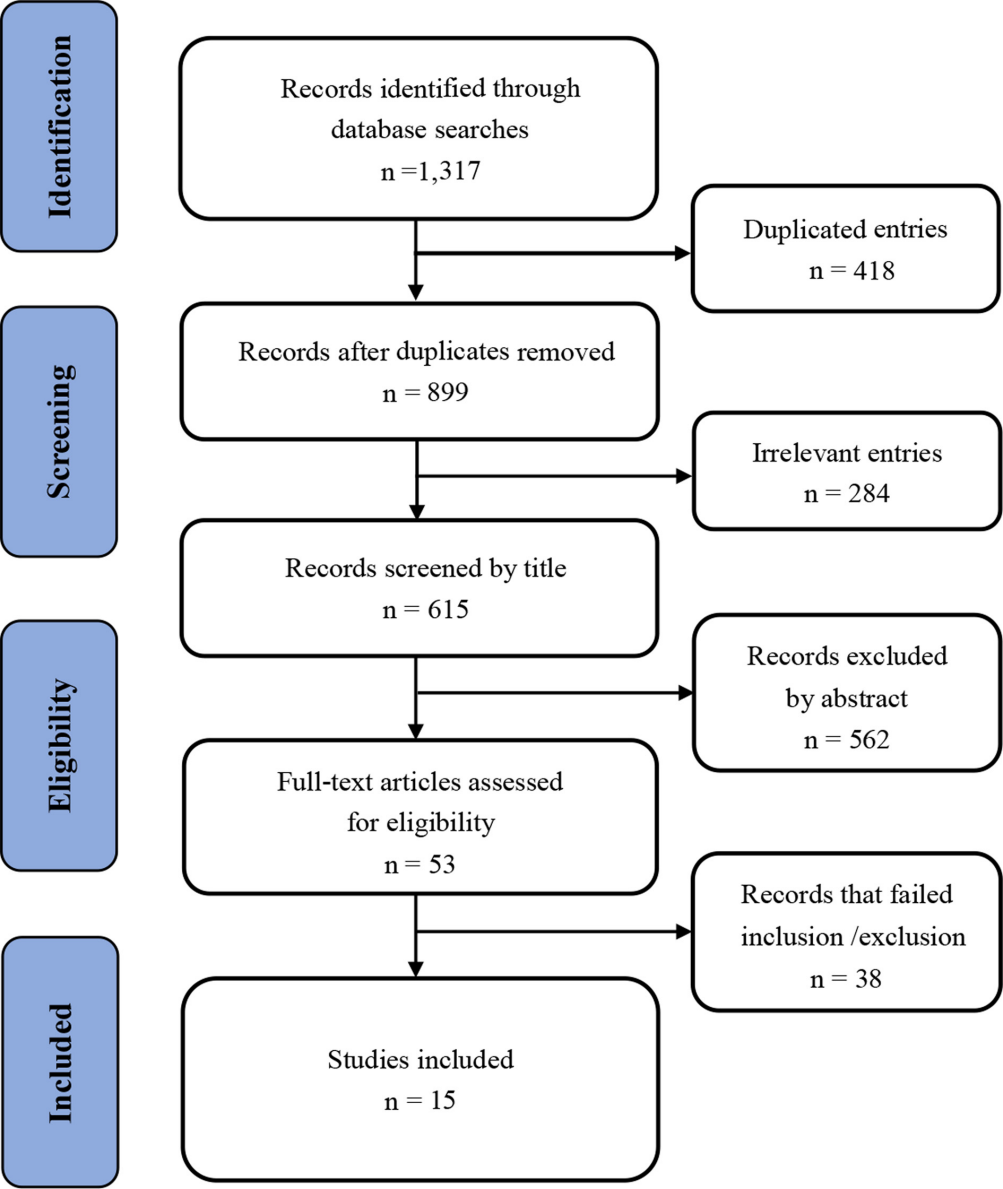
Flow diagram of the article selection

A total of 1,317 articles were retrieved during our initial search. After electronically removing 702 duplicated articles and irrelevant studies, we excluded 562 studies based on the assessment of their titles and abstracts. Subsequently, 53 articles were evaluated in detail. Of these, 38 articles were excluded after reviewing the full text, 15 unique studies were ultimately included in the pooled analysis [10–12,17–28]. The detailed screening process was presented in Figure 1.

The main characteristics of the included studies are summarized in Table 1. A total of 2,502 ESCC patients underwent surgery were included in the 15 studies, which were published from 2002 to 2021. As to TAMs identification, 13 articles adopted CD68 [10,11,17–25,27,28], five articles adopted CD163 [12,19,20,25,26], and three articles adopted CD204 as a macrophage marker [12,19,21,25] to detected TAMs by immunohistochemistry. Ten studies investigated the role of TAMs in both TI and TS [11,17–20,23–28], one study only investigated TAMs in TI [21], and two studies only investigated TAMs in TS [12,22]. Moreover, there were 14 articles that reported OS data [10–12,17–24,26–28], and five articles that reported DFS data [19,22,24,25,28]. The NOS scores of these studies ranged from 4 to 8 (Table 1).

**Table 1:** Characteristics of studies included in the pooled analysis

Author	Country	Sample size	Male	Markers	Tissue distribution	Type	Stage	Outcome assessment	NOS
Koide (2002)	Japan	56	42	CD68	Tumor islet and stroma	ESCC	Unavailable	OS	4
Guo (2007)	China	137	103	CD68	Tumor islet and stroma	ESCC	I-IV	OS	8
Shigeoka (2013)	Japan	70	55	CD68, CD163, CD204	Tumor islet and stroma	ESCC	I-IV	OS, DFS	7
Sugimura (2015)	Japan	210	186	CD68, CD163	Tumor islet and stroma	ESCC	Unavailable	OS	5
Hatogai (2016)	Japan	196	160	CD68, CD204	Tumor islet	ESCC	I-IV	OS	6
Li (2016)	China	705	430	CD68	Tumor stroma	ESCC	I-IV	OS, DFS	5
Xu (2016)	China	138	102	CD68	Tumor islet and stroma	ESCC	II-III	OS	7
Zhu (2016)	China	220	70	CD68	Tumor islet and stroma	ESCC	II	OS, DFS	5
Hosono (2017)	Japan	70	NA	CD68, CD163, CD204	Tumor islet and stroma	ESCC	I-IV	DFS	7
Hu (2017)	China	100	68	CD163	Tumor islet and stroma	ESCC	I-IV	OS	6
Wang (2017)	China	100	117	CD68	Tumor islet and stroma	ESCC	I-IV	OS	6
Lu (2019)	China	200	116	CD68, PD1	NA	ESCC	I-IV	OS, DFS	7
Yamamoto (2020)	Japan	86	75	CD86, CD163, CD206	Tumor stroma	ESCC	I-IV	OS	8
Chen (2021)	China	114	82	CD68, PD1	NA	ESCC	I-IV	OS	8
Jiang (2021)	China	100	66	CD68, HLA-DR	Tumor islet and stroma	ESCC	I-IV	OS	7

Abbreviations: DFS, disease-free survival; ESCC, esophageal squamous cell carcinoma; OS, overall survival; NA, not available; NOS, Newcastle–Ottawa scale.

### Prognostic significance of CD68+ TAMs

**Figure 2:**
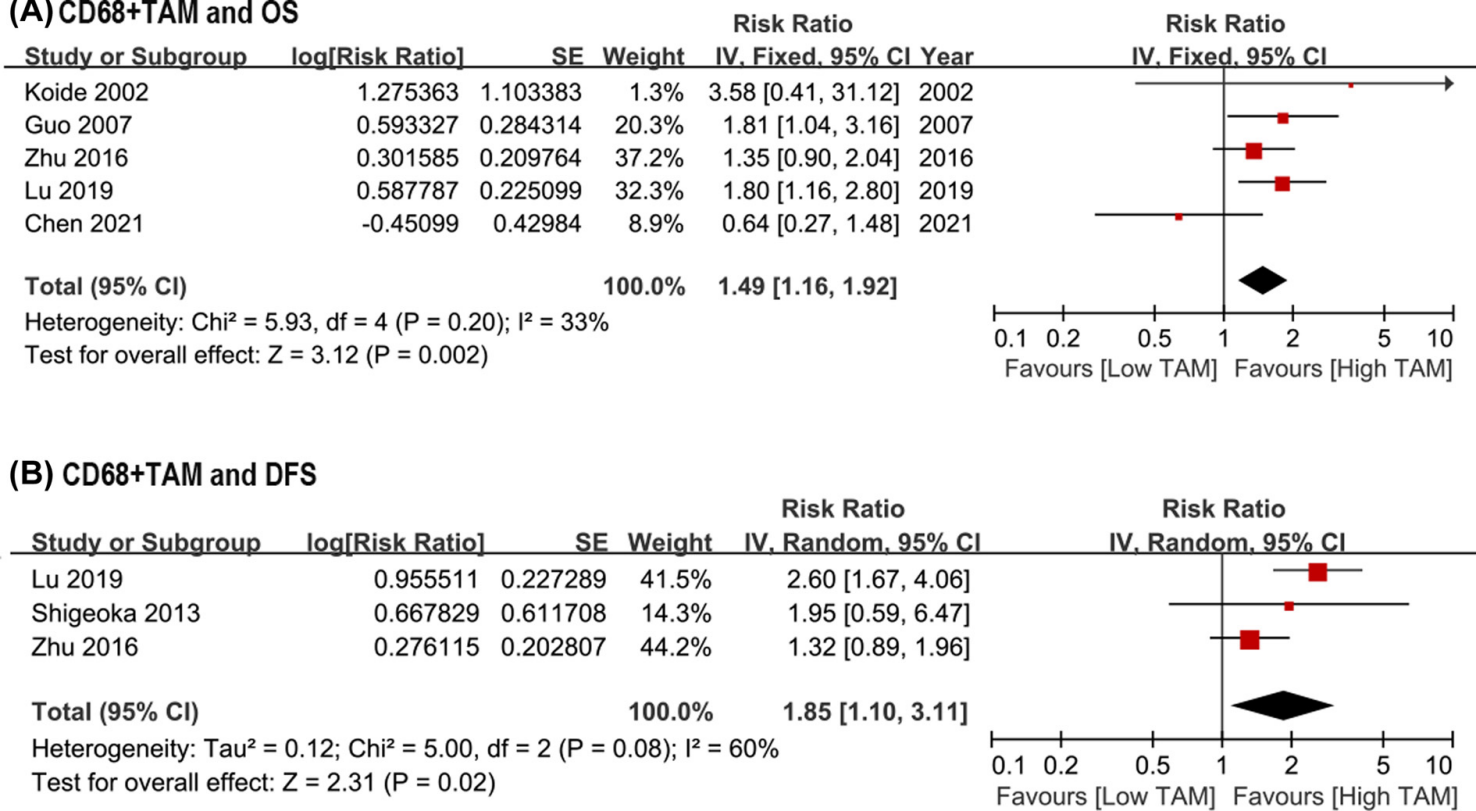
Forest plots of HR for survival outcomes between high and low density of total CD68+ TAMs infiltration in the tumor among ESCC patients underwent surgery. (**A**) HR of OS for total CD68+ TAMs in the tumor; (**B**) HR of DFS for total CD68+ TAMs in the tumor. DFS, disease-free survival; ESCC, esophageal squamous cell carcinoma; HR, hazard risk; OS, overall survival; TAM, tumor-associated macrophages.

A total of 13 studies were included in the analysis of the prognostic significance of CD68+ TAMs on survival outcomes in patients with ESCC. As for the total count of CD68+ TAMs, a high CD68+ TAMs density was significantly associated with poor OS (HR = 1.49, 95% CI: 1.16–1.92, *P*=0.002; *I*^2^ = 33%; Figure 2A) and DFS (HR = 1.85, 95% CI: 1.10–3.11, *P*=0.02; *I*^2^ = 60%; Figure 2B), compared with a low CD68+ TAMs density.

**Figure 3:**
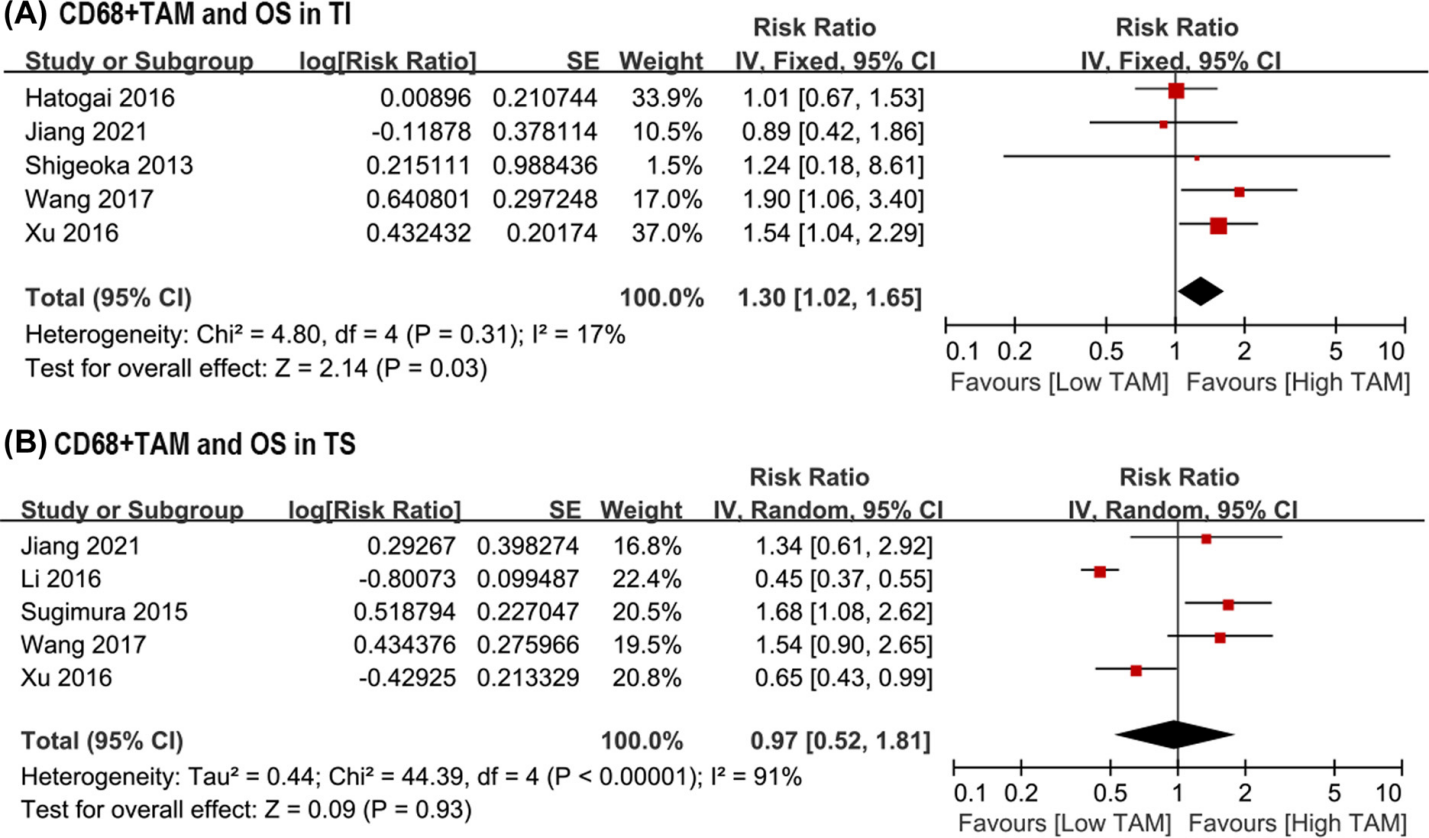
Forest plots of HR for OS between high and low density of CD68+ TAMs in ESCC patients underwent surgery. (**A**) HR of OS for CD68+ TAMs in TI; (**B**) HR of OS for CD68+ TAMs in TS. ESCC, esophageal squamous cell carcinoma; HR, hazard risk; OS, overall survival; TAM, tumor-associated macrophage; TI, tumor islet; TS, tumor stroma.

In addition, higher CD68+ TAMs density predicted worse OS than lower CD68+ TAMs density in TI, with a pooled HR of 1.30 (95% CI: 1.02–1.65, *P*=0.03; *I*^2^ = 17%; Figure 3A). However, the pooled HR showed that CD68+ TAMs infiltration in TS was not associated with OS (HR = 0.97, 95% CI: 0.52–1.81, *P*=0.93; *I*^2^ = 91%; Figure 3B).

### Prognostic significance of CD163+ TAMs

**Figure 4:**
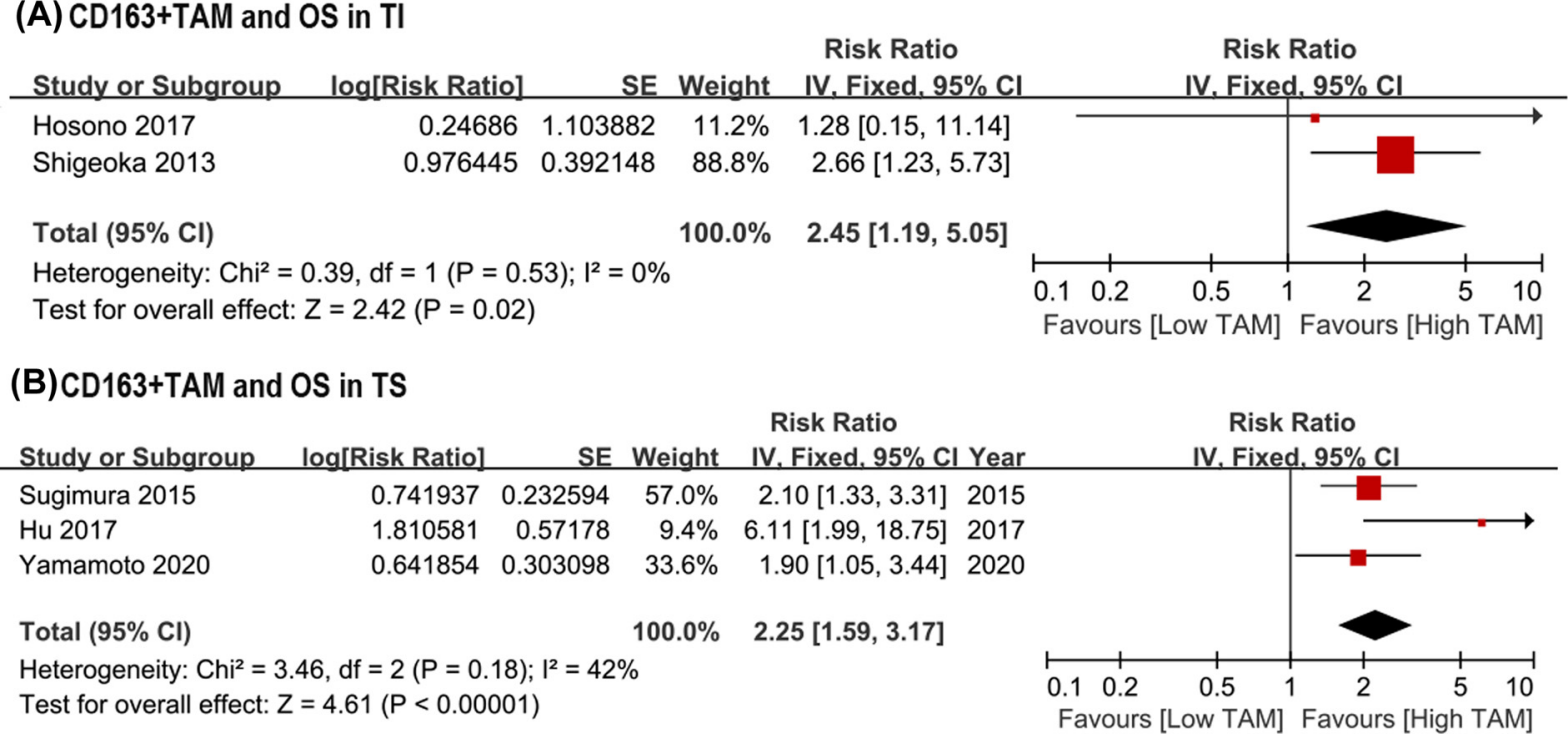
Forest plots of HR for OS between high and low density of CD163+ TAMs in ESCC patients underwent surgery. (**A**) HR of OS for CD163+ TAMs in TI; (**B**) HR of OS for CD163+ TAMs in TS. ESCC, esophageal squamous cell carcinoma; HR, hazard risk; OS, overall survival; TAM, tumor-associated macrophage; TI, tumor islet; TS, tumor stroma.

To assess the role of CD163+ TAMs on survival outcomes in patients with ESCC, a total of five studies were included in the analysis. This pooled analysis was performed in fixed-effect model for the low heterogeneity in the followed results (*I^2^* < 50%). Relative to a high density of CD163+ TAMs, a low density of CD163+ TAMs indicated better OS in TI (HR = 2.45, 95% CI: 1.19–5.05, *P*=0.02; *I*^2^ = 0; Figure 4A), which was similar to CD163+ TAMs in TS (HR = 2.25, 95% CI: 1.59–3.17, *P*<0.00001; *I*^2^ = 42%; Figure 4B).

### Prognostic significance of CD204+ TAMs

**Figure 5:**
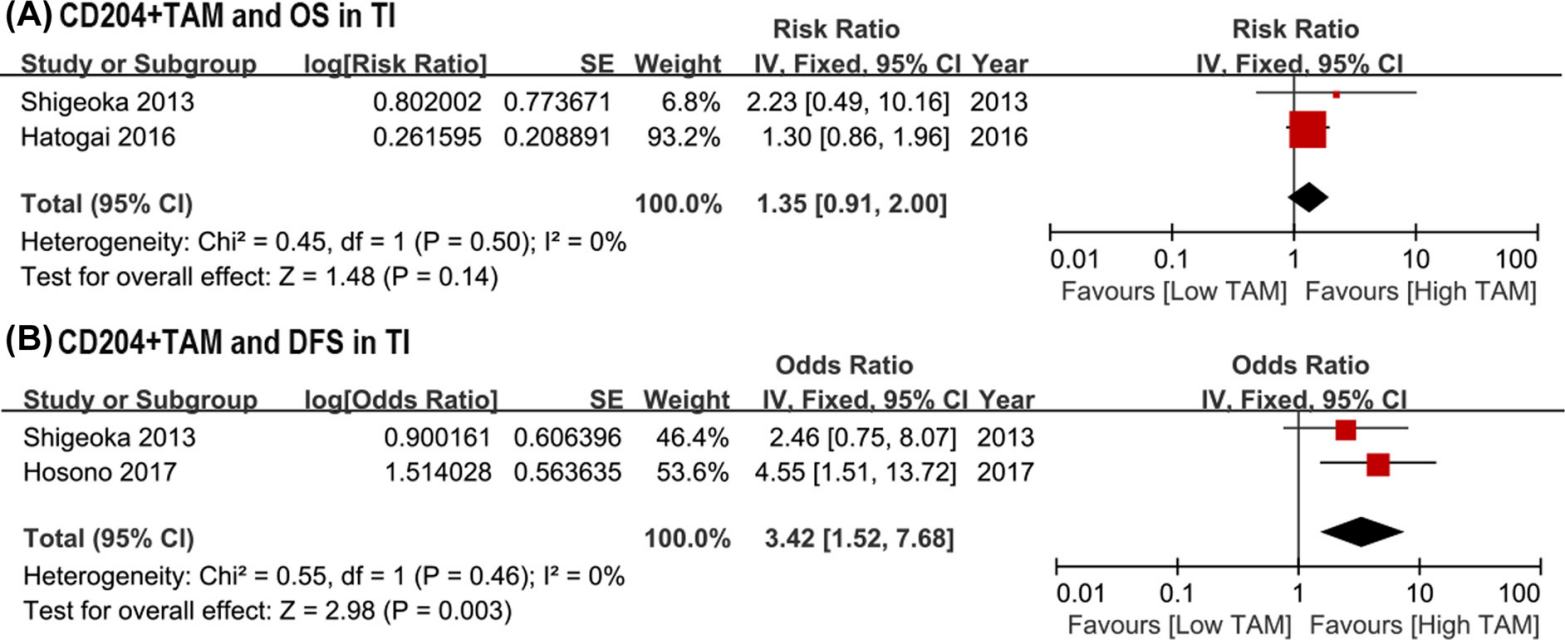
Forest plots of HR for survival outcomes between high and low density of CD204+ TAMs in TI among ESCC patients underwent surgery. (**A**) HR of OS for CD204+ TAMs in TI; (**B**) HR of DFS for CD204+ TAMs in TI. DFS, disease-free survival; ESCC, esophageal squamous cell carcinoma; HR, hazard risk; OS, overall survival; TAM, tumor-associated macrophage; TI, tumor islet.

Given the absent of heterogeneity, the fixed-effect model was used in assessing the association between CD204+ TAMs and survival outcomes (*I*^2^ = 0), despite only three studies included. The pooled analysis evaluating the role of CD204+ TAMs on OS showed no statistically significant differences in TI (HR = 1.35, 95% CI: 0.91–2.00, *P*=0.14; *I*^2^ = 0; Figure 5A), but higher 204+ TAMs density indicated worse DFS compared with lower 204+ TAMs density in TI (HR = 3.42, 95% CI: 1.52–7.68, *P*=0.003; *I*^2^ = 0; Figure 5B)

### Association between TAMs and clinicopathological characteristics

We also analyzed the association between TAMs (CD68+ or CD163+) and clinicopathological characteristics in ESCC patients who underwent surgery. Unlike sex, histologic grade, and T grade, the pooled results suggested that a high density of total CD68+ TAMs was significantly associated lymphatic vessel invasion (OR = 2.55, 95% CI: 1.43–4.54, *P*=0.001; *I*^2^ = 0), vascular invasion (OR = 3.25, 95% CI: 1.94–5.47, *P*<0.00001; *I*^2^ = 29%), and lymph node metastasis (OR = 1.85, 95% CI: 1.05–3.28, *P*=0.03; *I*^2^ = 20%). Especially, a high CD68+ TAMs density had a significant association with lymph node metastasis in TI (OR = 2.10, 95% CI: 1.19–3.71, *P*=0.01; *I*^2^ = 0) or TS (OR = 1.58, 95% CI: 1.00–2.51, *P*=0.05; *I*^2^ = 0). However, the results in TI did not reveal any significant association between CD163+ TAMs and any clinicopathological characteristics, including histologic grade and lymph node metastasis (Table 2).

**Table 2:** Association between TAMs and clinicopathological characteristics of ESCC patients underwent surgery

Clinicopathological characteristics	References	No. of studies	Model	Pooled OR (95% CI)	*P-*value	Heterogeneity	
						*I*^2^ (%)	*P-*value
**CD68+ TAMs (TI+TS)**							
Sex (males vs. females)	Males	4	Fixed	0.89 (0.60–1.33)	0.58	0	0.39
Histologic grade (poor vs. well-moderate)	Poor	3	Fixed	1.21 (0.60–2.45)	0.60	30	0.24
Lymphatic vessel invasion (positive vs. negative)	Positive	2	Fixed	2.55 (1.43–4.54)	0.001	0	0.72
Vascular invasion (positive vs. negative)	Positive	3	Fixed	3.25 (1.94–5.47)	< 0.00001	29	0.24
T grade (T2-4 vs. T1)	T2-4	2	Random	1.23 (0.15–10.10)	0.85	93	< 0.001
Lymph node metastasis (positive vs. negative)	Positive	2	Fixed	1.85 (1.05–3.28)	0.03	20	0.26
**CD68+ TAMs (TI)**							
Lymphatic vessel invasion (positive vs. negative)	Positive	2	Fixed	2.10 (1.19–3.71)	0.01	0	0.38
**CD68+ TAMs (TS)**							
Age (≥60 y vs. <60 y)	≥60 years	2	Fixed	1.13 (0.71–1.80)	0.60	0	0.98
Sex (males vs. females)	Males	3	Fixed	0.79 (0.51–1.23)	0.30	0	0.92
Location of the primary tumor (upper vs. middle + lower)	Upper	2	Fixed	1.20 (0.63–2.31)	0.58	0	0.90
Histologic grade (poor vs. well-moderate)	Poor	2	Random	1.40 (0.54–3.59)	0.49	74	0.05
T grade (T3-4 vs. T1-2)	T3-4	2	Random	1.94 (0.47–8.01)	0.36	89	0.003
Lymph node metastasis (positive vs. negative)	Positive	2	Fixed	1.58 (1.00–2.51)	0.05	0	0.86
p-stage (III-IV vs. I-II)	III-IV	2	Random	1.46 (0.72–2.95)	0.30	50	0.16
**CD163+ TAMs (TI)**							
Histologic grade (poor vs. well-moderate)	Poor	2	Fixed	1.23 (0.58–2.59)	0.59	0	0.41
Lymph node metastasis (positive vs. negative)	Positive	2	Fixed	1.73 (0.95–3.15)	0.07	0	0.43

Abbreviations: CI, confidence interval; ESCC, esophageal squamous cell carcinoma; OR, odds ratio; TAM, tumor-associated macrophage; TI, tumor islet; TS, tumor stroma.

### Sensitivity analysis and publication bias

**Figure 6:**
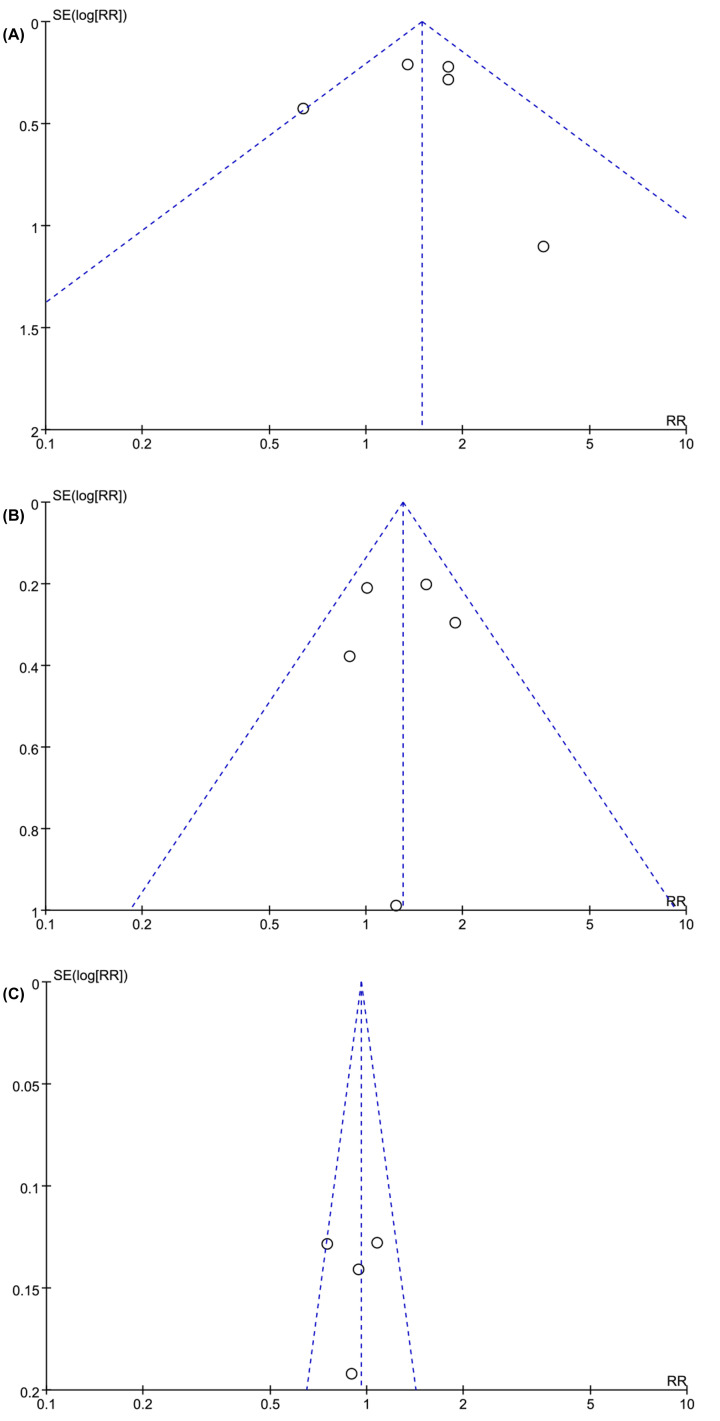
Funnel plot of studies with TAM density for potential publication bias assessment. (**A**) OS and CD68+ TAMs in the tumor; (**B**) OS and CD68+ TAMs in TI; (**C**) OS and CD68+ TAMs in TS. OS, overall survival; TAM, tumor-associated macrophage; TI, tumor islet; TS, tumor stroma.

Our analyses were robust in terms of choice of the models and statistical methods. The relationship between total CD68+TAMs and OS did not alter by shifting a fixed-effect model to a random-effect model (HR = 1.47, 95% CI: 1.05–2.04, *P*=0.02). According to the funnel plot of the standard error by log OR, there was no significant publication bias in the present pooled analysis (Figure 6).

## Discussion

Because of the insidious onset, the diagnosis of ESCC is usually delayed, resulting in rapid invasion and challenging treatment [29]. Currently, therapeutic approaches for ESCC consist mainly of surgery, chemotherapy and radiotherapy, with high recurrence rates and fatality rates [2]. TAMs, an important component in tumor microenvironment, might become a promising direction for ESCC therapy. The recent ongoing experimental and pre-clinical TAM-targeted studies have shown that TAMs are connected with prognosis in ESCC patients, yielding different conclusions. Therefore, we conducted a comprehensive analysis to investigate the association between TAMS (CD68+, CD163+ or CD204+) and survival outcomes (OS or DFS) in ESCC through pooling data from 2,502 patients who underwent surgery.

In this pooled analysis, a total of 15 studies were included to analyze the prognostic and clinical significance of TAMs in ESCC patients who underwent surgery. Among these studies, 13 studies used CD68 as a biomarker for TAM identification in tumor tissue, while five and three studies used CD163 and CD204, respectively. Our results suggested that a high CD68+ TAMs density in the tumor microenvironment was significantly associated with poor prognosis (OS and DFS) than a low CD68+ TAMs density. Similarly, greater CD68+ TAMs density in TI predicted worse OS, although no significant association was observed between CD68+ TAMs in TS and OS. Moreover, higher CD163+ TAMs density indicated worse OS in TI and TS. Unlike no statistical significance between CD204+ TAMs and OS, a high density of CD204+ TAMs predicted poor DFS in TI. In addition, we also analyzed the association between TAMs and clinicopathological characteristics in ESCC patients who underwent surgery, which demonstrated that a high density of CD68+ TAMs was significantly associated with lymphatic vessel invasion, vascular invasion, and lymph node metastasis. However, it is important to note that there is substantial heterogeneity, necessitating further studies with larger sample size to validate these conclusions.

From an oncological viewpoint, TAMs have been commonly polarized into two distinct macrophage phenotypes: pro-inflammatory M1 (classically activated macrophages) with tumor suppressive capabilities, and anti-inflammatory M2 (alternatively activated macrophages) with tumor supportive capabilities [12]. M2 macrophages, which contribute to the progression of ESCC by promoting tumor cell growth, invasion and metastasis, as well as restraining anti-tumor immune response cells [30], are characterized by the specific receptors known as CD163 (hemoglobin scavenger receptor) and CD204 (macrophage scavenger receptor I) [31,32]. This may explain that greater CD163+ and CD204+ TAMs density suggested worse prognosis. However, because of the limited studies included in our analysis, we did not find a significant association between CD204+ TAM density in TI and OS. As a biomarker used to identify TAMs, CD68 has been widely accepted by scholars. In our pooled analysis, 13 out of 15 included studies used CD68 for TAM identification. Although the total CD68+ TAMs infiltration in the tumor was significantly associated with poor OS, the survival outcomes of CD68+ TAMs in TI and TS were inconsistent. It has been reported that TAMs infiltrating in TI might be the M2 type predominantly, while TAMs infiltrating in TS might display characteristics of the M1 type [23,27]. This could be the reason why greater CD68+ TAMs density in TI predicted worse OS. However, no significant association was observed between CD68+ TAMs in TS and OS, owing to the attainable limited data.

For avoiding the confounding factors as possible as we can, this pooled analysis exclusively enrolled ESCC patients who underwent surgery. Moreover, we have extensively incorporated studies examining the prognostic and clinical significance of TAMs in ESCC, to make our findings more reliable. Besides, the sensitivity analysis was robust, and no publication bias was detected, which ensured the validity of the present results. Finally, the subgroup analyses were conducted according to different TAMs markers (CD68, CD163 and CD204) and histologic locations (TI+TS, TI, and TS) to assess the effect of TAMs on the prognosis of ESCC, including OS and DFS, which made our pooled analysis more informative and persuasive.

In addition to these strengths, several limitations are existing in our study. On the one hand, the pooled analysis was performed in meticulous detail to minimize heterogeneity, including distinct TAMs markers, different histologic locations, as well as OS or DFS; therefore, there was not enough of such information available from the included studies to perform a pooled analysis, such as HLA-DR+ TAMs, M1 and M2 TAMs. On the other hand, all included studies were retrospective study, which might result in the selection bias in our results. Furthermore, the heterogeneity was significant in some pooled analyses, we still should use caution when drawing conclusions, although a random effects model was used on account of this heterogeneity.

## Conclusion

This study represents the first comprehensive analysis of the prognostic and clinical significance of TAMs in ESCC, using existing literature. The findings of our pooled analysis suggested that the higher density of total CD68+ TAMs infiltration in the tumor indicated poor OS and DFS, while greater CD163+ and CD204+ TAMs density suggested worse prognosis in ESCC patients underwent surgery, Moreover, a high CD68+ TAMs density was closely link to lymphatic vessel invasion, vascular invasion, and lymph node metastasis. Given the limitations, further large-scale studies are still required to confirm the conclusion in our study.

## Data Availability

The datasets generated during and/or analyzed during the current study are available from the corresponding author on reasonable request.

## Abbreviations

CI: confidence interval
DFS: disease-free survival
ESCC: esophageal squamous cell carcinoma
HRs: hazard ratios
KM: Kaplan–Meier
NOS: Newcastle–Ottawa scale
OS: overall survival
PRISMA: Preferred Reporting Items for Systematic Reviews and Meta‐Analyses
TAM: tumor-associated macrophage
TI: tumor islet
TS: tumor stroma
